# Non-contiguous finished genome sequence and description of *Anaerococcus pacaensis* sp. nov., a new species of anaerobic bacterium

**DOI:** 10.4056/sigs.4177252

**Published:** 2013-08-10

**Authors:** Isabelle Pagnier, Olivier Croce, Catherine Robert, Didier Raoult, Bernard La Scola

**Affiliations:** 1 Unité de Recherche sur les Maladies Infectieuses et Tropicales Emergentes Faculté de médecine, Aix-Marseille Université, Marseille, France

**Keywords:** *Anaerococcus pacaensis*, genome

## Abstract

*Anaerococcus pacaensis* strain 9403502^T^, is the type strain of *Anaerococcus pacaensis* sp. nov., a new species within a new genus *Anaerococcus*. This strain, whose genome is described here, was isolated from a blood sample. *A. pacaensis* strain 9403502^T^ is an obligate anaerobic Gram-positive coccus. Here we describe the features of this organism, together with the complete genome sequence and annotation. The 2.36 Mbp long genome exhibits a G+C content of 35.05% and contains 2,186 protein-coding and 72 RNA genes, including 3 rRNA genes.

## Introduction

*Anaerococcus pacaensis* strain 9403502^T^ (= CSUR P122 = DSM 26346), is the type strain of *Anaerococcus pacaensis* sp. nov., and a member of the genus *Anaerococcus*. This bacterium is a Gram-positive, anaerobic, non spore-forming, indole negative coccus that was isolated from a blood sample, during a study prospecting anaerobic isolates from deep samples [1].

The “gold standard” method to define a new bacterial species or genus is DNA-DNA hybridization and G+C content determination [2]. Those methods are expensive and poorly reproducible and actually, bacterial species can be classified with PCR and sequencing methods, particularly 16S rRNA sequences with internationally-validated cutoff [3]. More recently, an increasing number new bacterial genera and species have been described using high throughput genome sequencing and mass spectrometric analyses that allow access to the wealth of genetic and proteomic information [4,5]. In the past, studies have described new bacterial species and genera using genome sequencing, MALDI-TOF spectra, main phenotypic characteristics [6-23], and we propose here to describe a new species within the genus *Anaerococcus* in the same way.

Here we present a summary classification and a set of features for *A. pacaensis* sp. nov. strain 9403502^T^ (= CSUR P122= DSM 26346) together with the description of the complete genomic sequencing and annotation. These characteristics support the circumscription of a novel species, *Anaerococcus pacaensis* sp. nov., within the genus *Anaerococcus*, and within the *Clostridiales* Family XI *Incertae sedis*.

The genus *Anaerococcus* was first described in 2001 [24], and belongs to the *Clostridiales* Family XI *Incertae sedis*. This family is defined mainly on the basis of phylogenetic analyses of ARNr 16S sequences, and in the *Anaerococcus* genus, bacteria are all anaerobic gram positive cocci. Based on the comparison of the 16S rRNA gene sequence, the first closest related species to *Anaerococcus pacaensis* sp., nov., is *Anaerococcus prevotii*. It was first described in 1948 by Foubert and Douglas [25] and reclassified later in the genus *Anaerococcus* [24]. The second closest related species is *A. octavius*, which was described first as *Peptostreptococcus octavius*, isolated from a human sample in 1998 by Murdoch et al [26]. It was later re-classified in the genus *Anaerococcus*, as *A. octavius* [24].

## Classification and features

**Figure 1:**
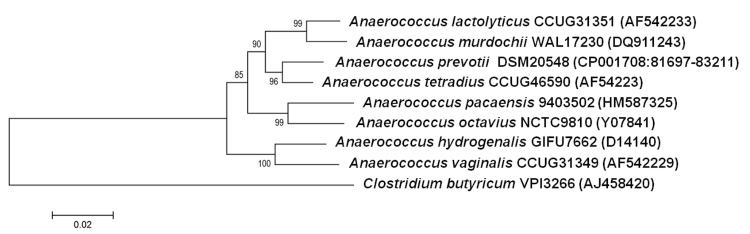
Phylogenetic tree highlighting the position of *Anaerococcus pacaensis* strain 9403502^T^ relative to other type strains within the genus *Anaerococcus.* GenBank accession numbers are indicated in parentheses. Sequences were aligned using CLUSTALW, and phylogenetic inferences obtained using the maximum-likelihood method within the MEGA 4 software [39]. Numbers at the nodes are bootstrap values obtained by repeating the analysis 500 times the analysis to generate a majority consensus tree. *Clostridium butyricum* was used as outgroup. The scale bar represents a 2% nucleotide sequence divergence.

A blood sample was collected from a patient during a study analyzing emerging anaerobes, with MALDI-TOF and 16S rRNA gene sequencing [1]. The specimen was sampled in Marseille and preserved at -80°C after collection. Strain 9403502^T^ (Table 1) was isolated in July 2009, by anaerobic cultivation on 5% sheep blood-enriched Columbia agar (BioMerieux, Marcy l’Etoile, France). This strain exhibited a 95% nucleotide sequence similarity with *Anaerococcus prevotii* [24,25]. Those similarity values are lower than the threshold recommended to delineate a new genus without carrying out DNA-DNA hybridization [38]. In the inferred phylogenetic tree, it forms a distinct lineage close to *A. octavius* (Figure 1).

**Table 1:** Classification and general features of *Anaerococcus pacaensis* strain 9403502^T^

**MIGS ID**	**Property**	**Term**	**Evidence code^a^**
		Domain *Bacteria*	TAS [27]
		Phylum *Firmicutes*	TAS [28-30]
		Class *Clostridia*	TAS [31,32]
	Current classification	Order *Clostridiales*	TAS [33,34]
		Family XI *Incertae sedis*	TAS [35]
		Genus *Anaerococcus*	TAS [36]
		Species *Anaerococcus pacaensis*	IDA
		Type strain 9403502^T^	IDA
	Gram stain	Positive	IDA
	Cell shape	Cocci	IDA
	Motility	Non motile	IDA
	Sporulation	Non spore-forming	IDA
	Temperature range	Mesophile	IDA
	Optimum temperature	37°C	IDA
MIGS-6.3	Salinity	Weak growth on BHI medium + 1% NaCl	IDA
MIGS-22	Oxygen requirement	Anaerobic	IDA
	Carbon source	Unknown	NAS
	Energy source	Unknown	NAS
MIGS-6	Habitat	Blood	IDA
MIGS-15	Biotic relationship	Free living	IDA
MIGS-14	Pathogenicity Biosafety level Isolation	Unknown 2 Human blood sample	NAS
MIGS-4	Geographic location	France	IDA
MIGS-5	Sample collection time	July 2009	IDA
MIGS-4.1	Latitude	43.296482	IDA
MIGS-4.1	Longitude	5.36978	IDA
MIGS-4.3	Depth	Surface	IDA
MIGS-4.4	Altitude	0 above see level	IDA

**^a^**Evidence codes - IDA: Inferred from Direct Assay; TAS: Traceable Author Statement (i.e., a direct report exists in the literature); NAS: Non-traceable Author Statement (i.e., not directly observed for the living, isolated sample, but based on a generally accepted property for the species, or anecdotal evidence). These evidence codes are from the Gene Ontology project [37]. If the evidence is IDA, then the property was directly observed for a live isolate by one of the authors or an expert mentioned in the acknowledgements.

Different growth temperatures (23°C, 25°C, 28°C, 32°C, 35°C, 37°C, 50°C) were tested; no growth occurred at 23°C, 25°C, 28°C and 50°C, growth occurred between 32° and 37°C, and optimal growth was observed at 37°C.

**Figure 2:**
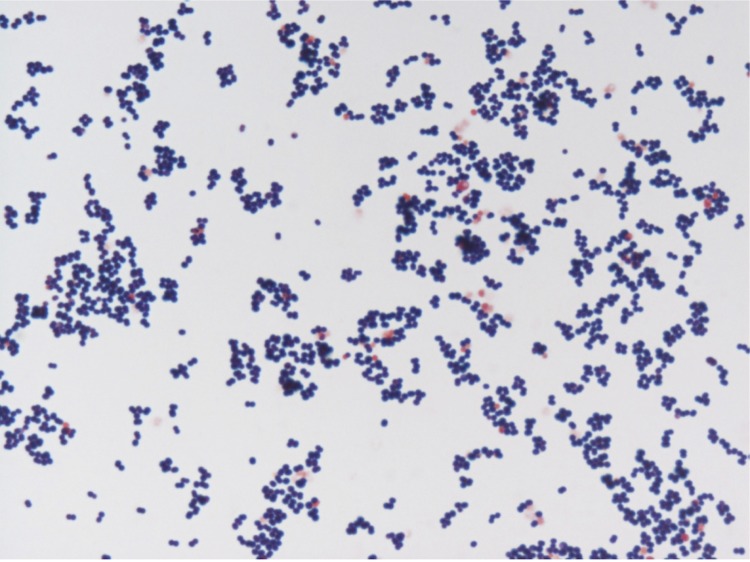
Gram staining of *A. pacaensis* strain 9403502^T^

**Figure 3:**
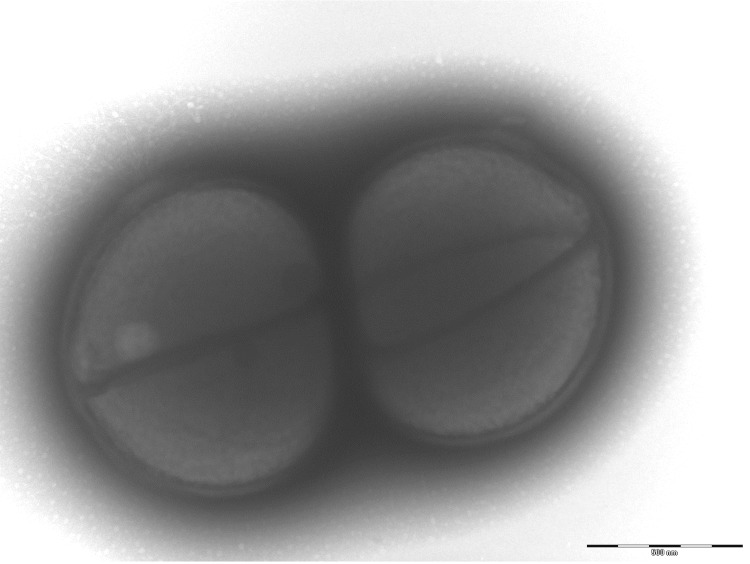
Transmission electron microscopy of *A. pacaensis* strain 9403502^T^, using a Morgani 268D (Philips) at an operating voltage of 60kV. The scale bar represents 500 nm.

Colonies are punctiform, very small, grey, dry and round on blood-enriched Columbia agar under anaerobic conditions using GENbag anaer (BioMérieux). Bacteria were grown on blood-enriched Columbia agar (Biomerieux), in BHI broth medium, and in Trypticase-soja TS broth medium, under anaerobic conditions using GENbag anaer (BioMérieux), under microaerophilic conditions using GENbag microaer (BioMérieux) and in the presence of air, with 5%CO_2_. They also were grown under anaerobic conditions on BHI agar, and on BHI agar supplemented with 1% NaCl. Growth was achieved only anaerobically, on blood-enriched Columbia agar, and weakly on BHI agar, and BHI agar supplemented with 1% NaCl after 72h incubation. Gram staining showed round non spore-forming Gram-positive cocci (Figure 2). The motility test was negative. Cells grow anaerobically in TS broth medium have a mean diameter of 1.140µm (min = 0.955µm; max = 1.404µm), as determined using electron microscopic observation after negative staining (Figure 3).

Strain 9403502^T^ exhibited catalase activity but no oxidase activities. Using API 20A, a positive reaction could be observed only weekly for Gelatinase. Using Api Zym, a positive reaction was observed for alkaline phosphatase (5nmol of hydrolyzed substrata), acid phosphatase (5nmol), naphtolphosphohydrolase (5nmol), and hyaluronidase (40nmol). Using Api rapid id 32A, a positive reaction could be observed only for beta glucuronydase and pyroglutamic acid arylamidase. Regarding antibiotic susceptibility, *A. pacaensis* was susceptible to penicillin G, amoxicillin, cefotetan, imipenem, metronidazole and vancomycin. When compared to the representative species within the genus *Anaerococcus*, *A. pacaensis* exhibits the phenotypic characteristics details in Table 2 [40].

**Table2:** Differential characteristics of *Anaerococcus pacaensis* sp. nov., strain 9403502^T^, *A. octavius* strain NCTC 9810^T^, and *A. tetradius* strain DSM 2951^T^.

**Properties**	*A. pacaensis*	*A. octavius*	*A. tetradius*
Cell diameter (µm)	0.9-1.4	0.7-0.9	0.5-1.8
Oxygen requirement	Anaerobic	Anaerobic	Anaerobic
Gram stain	Positive	Positive	Positive
Optimal growth temperature	37°C	na	na
Habitat	Human	Human	Human
			
**Enzyme production**			
Indole	-	-	-
Alkaline Phosphatase	+	-	-
Urease	-	-	+
Catalase	+	-	-
Gelatinase	+	na	na
			
**Activity of**			
Phosphatase	Acid phosphatase	na	na
	Naphtolphosphohydrolase		
			
Saccharolytic enzyme	Hyaluronidase	-	α-glucosidase
			ß-glucosidase
			ß-glucuronidase
			
Proteolytic enzyme	-	Proline arylamidase	Arginine arylamidase
		Pyroglutamyl arylamidase	Histidine arylamidase
			
**Utilization of**			
Glucose	-	+	+
Mannose	-	+	+
Lactose	-	-	-
Raffinose	-	-	+

**Figure 4:**
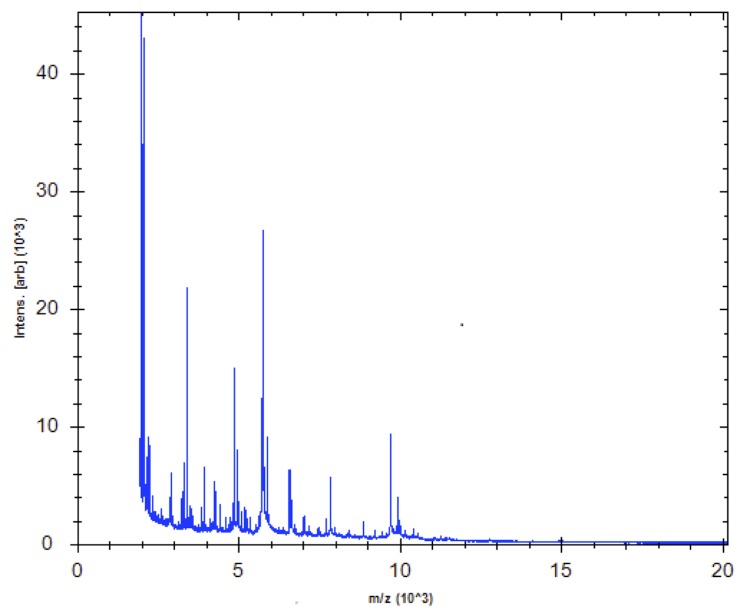
Reference mass spectrum from *A. pacaensis* strain 9403502^T^. Spectra from 10 individual colonies were compared and a reference spectrum was generated.

**Figure 5:**
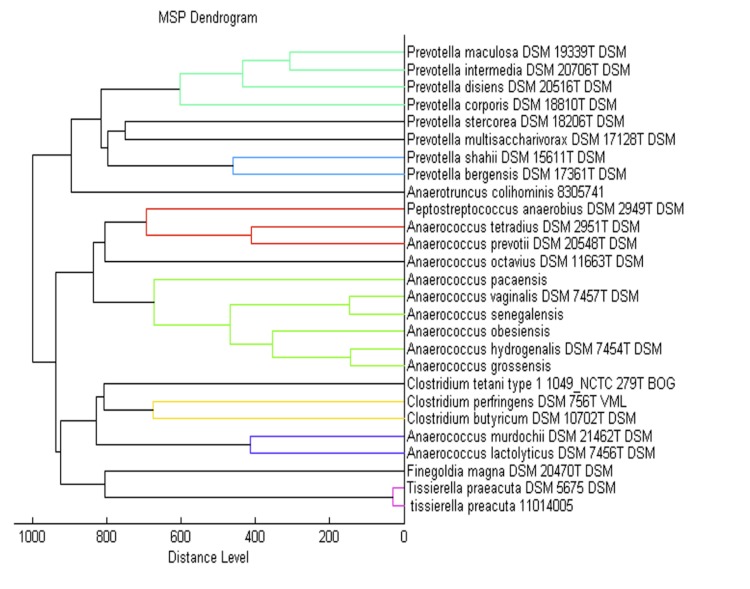
Dendrogram based on the comparison of the *A. pacaensis* strain 9403502^T^ MALDI-TOF reference spectrum, and 26 other species of the order of *Clostridiales*.

Matrix-assisted laser-desorption/ionization time-of-flight (MALDI-TOF) MS protein analysis was carried out as previously described [41]. A pipette tip was used to pick one isolated bacterial colony from a culture agar plate, and to spread it as a thin film on a MTP 384 MALDI-TOF target plate (Bruker Daltonics, Germany). Ten distinct deposits were done for strain 9403502^T^ from ten isolated colonies. Each smear was overlaid with 2 µL of matrix solution (saturated solution of alpha-cyano-4-hydroxycinnamic acid) in 50% acetonitrile, 2.5% tri-fluoracetic acid, and allowed to dry for five minutes. Measurements were performed with a Microflex spectrometer (Bruker). Spectra were recorded in the positive linear mode for the mass range of 2,000 to 20,000 Da (parameter settings: ion source 1 (ISI), 20kV; IS2, 18.5 kV; lens, 7 kV). A spectrum was obtained after 675 shots at a variable laser power. The time of acquisition was between 30 seconds and 1 minute per spot. The ten 9403502^T^ spectra were imported into the MALDI Bio Typer software (version 2.0, Bruker) and analyzed by standard pattern matching (with default parameter settings) against the main spectra of 5,697 bacteria, in the Bio Typer database. The method of identification includes the m/z from 3,000 to 15,000 Da. For every spectrum, 100 peaks at most were taken into account and compared with the spectra in database. A score enabled the identification, or not, from the tested species: a score ≥ 2 with a validated species enabled the identification at the species level; a score ≥ 1.7 but < 2 enabled the identification at the genus level; and a score < 1.7 did not enable any identification. For strain 9403502^T^, the best obtained score was 1.265, which is not significant, suggesting that our isolate was not a member of a known genus. Our database was incremented with the reference spectrum from strain 9403502^T^ (Figure 4). A dendrogram was constructed with the MALDI Bio Typer software (version 2.0, Bruker), comparing the reference spectrum of strain 9403502^T^ with reference spectra of 26 bacterial species, all belonging to the order of *Clostridiales*. In this dendrogram, strain 9403502^T^ appears as a separated branch within the genus *Anaerococcus* (Figure 5).

## Genome sequencing and annotation

### Genome project history

The organism was selected for sequencing on the basis of its phylogenetic position, 16S rRNA similarity to other members of the *Anaerococcus* genus, and is part of a study prospecting anaerobic bacteria in several clinical deep samples. It was the first genome of the new genus *Anaerococcus pacaensis* sp. nov., and the 7th genome of *Anaerococcus sp.*

The Genbank accession number is CAJJ020000000 (CAJJ020000001-CAJJ020000053) and consists of 14 scaffolds with a total of 53 contigs. Table 2 shows the project information and its association with MIGS version 2.0 compliance.

### Growth conditions and DNA isolation

*A. pacaensis* sp. nov. strain 9403502^T^, CSUR= P122, DSM = 26346, was grown on blood agar medium at 37°C under anaerobic conditions. Eight petri dishes were spread and resuspended in 5 ×100µl of G2 buffer. A first mechanical lysis was performed by glass powder on the Fastprep-24 device (Sample Preparation system) from MP Biomedicals, USA during 2x20 seconds. DNA was then incubated for a lysozyme treatment (30 minutes at 37°C) and extracted through the BioRobot EZ 1 Advanced XL (Qiagen). The DNA was then concentrated and purified on a Qiamp kit (Qiagen). The yield and the concentration were measured by the Quant-it Picogreen kit (Invitrogen) on the Genios_Tecan fluorometer at 15.7ng/µl.

### Genome sequencing and assembly

A 3 kb paired end libraries was pyrosequenced on the 454 Roche Titanium. This project was loaded on a 1/4 region on PTP Picotiterplates. 5 µg of DNA was mechanically fragmented on the Hydroshear device (Digilab, Holliston, MA,USA) with an enrichment size at 3-4kb. The DNA fragmentation was visualized through the Agilent 2100 BioAnalyzer on a DNA labchip 7,500 with an optimal size of 3.2 kb. The library was constructed according to the 454 Titanium paired end protocol and manufacturer. Circularization and nebulization were performed and generated a pattern with an optimal at 604 bp. After PCR amplification through 15 cycles followed by double size selection, the single stranded paired end library was then quantified on the Agilent 2100 BioAnalyzer on a RNA pico 6,000 labchip at 91pg/µL . The library concentration equivalence was calculated at 2.76E+08 molecules/µL. The library was stocked at -20°C until using.

The library was clonal amplified with 0.5 and 1 cpb in 2 emPCR reactions in each condition with the GS Titanium SV emPCR Kit (Lib-L) v2 . The yield of the emPCR was 10.46 and 11.53% respectively according to the quality expected by the range of 5 to 20% from the Roche procedure. 790,000 beads were loaded on the GS Titanium PicoTiterPlates PTP Kit 70x75 sequenced with the GS Titanium Sequencing Kit XLR70.

The run was performed in overnight and then analyzed on the cluster through the gsRunBrowser and gsAssembler_Roche. The global 221,117 passed filter sequences generated 71.95Mb with a length average of 325bp.

The 454 sequencing generated 607,067 reads (105,03 Mb) assembled into contigs and scaffolds using Newbler version 2.7 (Roche) and Opera software v1.2 [42] combined to GapFiller V1.10 [43]. Finally, the available genome consists of 14 scaffolds and 53 contigs, with a coverage of 44.9.

### Genome annotation

Non-coding genes and miscellaneous features were predicted using RNAmmer [44], ARAGORN [45], Rfam [46], PFAM [47]. Open Reading Frames (ORFs) were predicted using Prodigal [48] with default parameters but the predicted ORFs were excluded if they were spanning a sequencing GAP region. The functional annotation was achieved using BLASTP [49] against the GenBank database [50] and the Clusters of Orthologous Groups (COG) database [51,52].

## Genome properties

**Figure 6:**
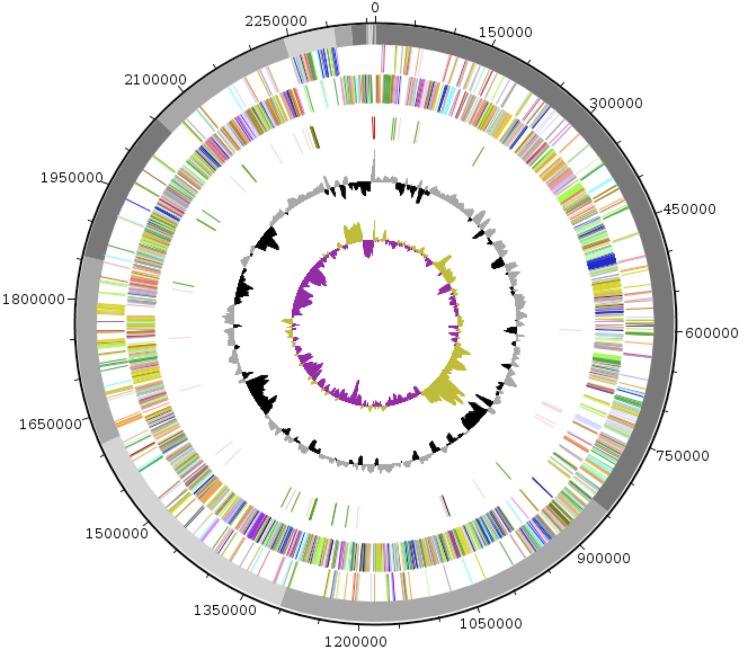
Graphical circular map of the genome. From outside to the center: scaffolds are in grey (unordered), genes on forward strand (colored by COG categories), genes on reverse strand (colored by COG categories), RNA genes (tRNAs green, rRNAs red, tm RNAs black, misc_RNA pink), GC content (black/grey), and GC skew (purple/olive).

The genome of *Anaerococcus pacaensis* strain 9403502^T^ is estimated at 2.36 Mb long with a G+C content of 35.05% (Figure 6 and Table 3). A total of 2,186 protein-coding and 72 RNA genes, including 3 rRNA genes, 42 tRNA, 1 tmRNA and 26 miscellaneous other RNA were founded. The majority of the protein-coding genes were assigned a putative function (74.1%) while the remaining ones were annotated as hypothetical proteins. The properties and the statistics of the genome are summarized in Tables 3 and 4. The Table 5 presents the difference of gene number (in percentage) related to each COG categories between *Anaerococcus pacaensis* and *Anaerococcus prevotii* DSM 20548. The proportion of COG is highly similar between the two species. The maximum difference is related to the COG "Carbohydrate Metabolism and transportation" which does not exceed 1.94%. The distribution of genes into COGs functional categories is presented in Table 6.

**Table 3:** Project information

**MIGS ID**	**Property**	**Term**
MIGS-31	Finishing quality	Non-contiguous finished
MIGS-28	Libraries used	One 454 PE 3-kb library
MIGS-29	Sequencing platforms	454 GS FLX+ Titanium
MIGS-31.2	Sequencing coverage	44.9
MIGS-30	Assemblers	Newbler 2.7
MIGS-32	Gene calling method	Prodigal 2.5
	Genbank ID	CAJJ020000000 (CAJJ020000001-CAJJ020000053)
	Genbank Date of Release	April 21, 2013
MIGS-13	Source material identifier	DSM 26346
	Project relevance	Prospection of anaerobic isolates in clinical samples
		

**Table 4:** Nucleotide content and gene count levels of the genome

Attribute	Value	% of Total
Genome size (bp)	2,360,033	100
DNA coding region (bp)	2,075,031	98.86
DNA G+C content (bp)	827,191	35.05
Total genes	2,272	100
rRNA	3	0.13
tRNA	42	1.85
tmRNA	1	0.04
miscRNA	26	1.14
Protein-coding genes	2,186	96.21
Genes with function prediction	1,620	74.10
Genes assigned to COGs	2,154	98.54

* The total is based on either the size of the genome in base pairs or the total number of protein coding genes in the annotated genome

**Table 5:** Number of genes associated with the 25 general COG functional categories

**Code**	**Value**	**% of total**^a^	**Description**
J	158	6.91	Translation
A	4	0.17	RNA processing and modification
K	160	6.99	Transcription
L	167	7.3	Replication, recombination and repair
B	4	0.17	Chromatin structure and dynamics
D	46	2.01	Cell cycle control, mitosis and meiosis
Y	0	0	Nuclear structure
V	97	4.24	Defense mechanisms
T	72	3.15	Signal transduction mechanisms
M	93	4.06	Cell wall/membrane biogenesis
N	16	0.7	Cell motility
Z	3	0.13	Cytoskeleton
W	0	0	Extracellular structures
U	48	2.1	Intracellular trafficking and secretion
O	93	4.06	Posttranslational modification, protein turnover, chaperones
C	129	5.64	Energy production and conversion
G	148	6.47	Carbohydrate transport and metabolism
E	145	6.34	Amino acid transport and metabolism
F	70	3.06	Nucleotide transport and metabolism
H	76	3.32	Coenzyme transport and metabolism
I	54	2.36	Lipid transport and metabolism
P	157	6.86	Inorganic ion transport and metabolism
Q	23	1.01	Secondary metabolites biosynthesis, transport and catabolism
R	272	11.89	General function prediction only
S	253	11.06	Function unknown
-	32	1.4	Not in COGs

^a^ The total is based on the total number of protein coding genes in the annotated genome.

**Table 6:** Percentage of genes associated with the 25 general COG functional categories for *Anaerococcus pacaensis* and *Anaerococcus prevotii* DSM 20548.

**Code**	**COG description**	***A. pacaensis*%age**	***A. prevotii* % age**	**Difference (in %)**
J	Translation	6.91	7.53	-0.62
A	RNA processing and modification	0.17	0.10	0.07
K	Transcription	6.99	6.91	0.08
L	Replication, recombination and repair	7.30	6.13	1.17
B	Chromatin structure and dynamics	0.17	0.16	0.01
D	Cell cycle control, mitosis and meiosis	2.01	1.56	0.45
Y	Nuclear structure	0.00	0.05	-0.05
V	Defense mechanisms	4.24	3.43	0.81
T	Signal transduction mechanisms	3.15	3.17	-0.02
M	Cell wall/membrane biogenesis	4.06	5.24	-1.18
N	Cell motility	0.70	0.36	0.34
Z	Cytoskeleton	0.13	0.16	-0.03
W	Extracellular structures	0.00	0.00	0.00
U	Intracellular trafficking and secretion	2.10	1.92	0.18
O	Posttranslational modification, protein turnover, chaperones	4.06	3.63	0.43
C	Energy production and conversion	5.64	6.59	-0.95
G	Carbohydrate transport and metabolism	6.47	8.41	-1.94
E	Amino acid transport and metabolism	6.34	6.65	-0.31
F	Nucleotide transport and metabolism	3.06	3.69	-0.63
H	Coenzyme transport and metabolism	3.32	3.58	-0.26
I	Lipid transport and metabolism	2.36	2.34	0.02
P	Inorganic ion transport and metabolism	6.86	6.80	0.06
Q	Secondary metabolites biosynthesis, transport and catabolism	1.01	0.78	0.23
R	General function prediction only	11.89	11.21	0.68
S	Function unknown	11.06	9.61	1.45
-	Not in COGs	1.40	0.99	0.41

## Insights into the genome sequence

We made some brief comparisons against *Anaerococcus prevotii* DSM 20548 (NC_013171), which is currently the closest available genome. This genome contains 1 chromosome (accession number: NC_013171) and 1 plasmid (accession number: NC_013164).

The draft genome sequence of *Anaerococcus pacaensis* has a bigger size compared to the *Anaerococcus prevotii* (respectively 2,36 Mbp and 1,99 Mbp). The G+C content is slightly larger than *Anaerococcus prevotii* too (respectively 37.5% and 35.05%). *Anaerococcus pacaensis* shares more genes (2,272 genes against 1,916 genes), however the ratios of genes per Mb is very similar (962,71 – 962,81).

## Conclusion

On the basis of phenotypic, phylogenetic and genomic analysis, we formally propose the creation of *Anaerococcus pacaensis*, whichcontains the strain 9403502^T^. This bacterium has been found in Marseille, France.

### Description of *Anaerococcus pacaensis* sp. nov.

*Anaerococcus pacaensis* (pa.ca’en.sis L. gen. masc. n. *pacaensis*, of PACA, the acronym of Provence Alpes Côte d’Azur, the region where was isolated *Anaerococcus pacaensis*). Isolated from a blood sample from a patient from Marseille. *A. pacaensis* is a Gram-positive cocci, obligate anaerobic, non-spore-forming bacterium. Grows on axenic medium at 37°C in anaerobic atmosphere. Negative for indole. Non-motile. The G+C content of the genome is 35.05%. The type strain is 9403502^T^ (= CSUR P122 = DSM 26346).

## References

[1] La Scola B Fournier PE Raoult D Burden of emerging anaerobes in the MALDI-TOF and 16S rRNA gene sequencing era. Anaerobe 2011; 17:106-112 10.1016/j.anaerobe.2011.05.010 21672636

[2] Rossello-Mora R. DNA-DNA Reassociation Methods Applied to Microbial Taxonomy and Their Critical Evaluation. *In*: Stackebrandt E (ed), Molecular Identification, Systematics, and population Structure of Prokaryotes. Springer, Berlin, 2006, p. 23-50.

[3] Stackebrandt E Ebers J Taxonomic parameters revisited: tarnished gold standards. Microbiol Today 2006; 33:152-155

[4] Welker M Moore ER Applications of whole-cell matrix-assisted laser-desorption/ionization time-of-flight mass spectrometry in systematic microbiology. Syst Appl Microbiol 2011; 34:2-11 10.1016/j.syapm.2010.11.013 21288677

[5] Tindall BJ Rosselló-Móra R Busse HJ Ludwig W Kämpfer P Notes on the characterization of prokaryote strains for taxonomic purposes. Int J Syst Evol Microbiol 2010; 60:249-266 10.1099/ijs.0.016949-0 19700448

[6] Kokcha S Michra AK Lagier JC Million M Leroy Q Raoult D Fournier PE Non-contiguous-finished genome sequence and description of *Bacillus timonensis* sp. nov. Stand Genomic Sci 2012; 6:346-355 10.4056/sigs.2776064 23408487 PMC3558959

[7] Lagier JC El Karkouri K Nguyen TT Armougom F Raoult D Fournier PE Non-contiguous-finished genome sequence and description of *Anaerococcus senegalensis* sp. nov. Stand Genomic Sci 2012; 6:116-125 10.4056/sigs.2415480 22675604 PMC3359877

[8] Mishra AK Gimenez G Lagier JC Robert C Raoult D Fournier PE Non-contiguous-finished genome sequence and description of *Alistipes senegalensis* sp. nov. Stand Genomic Sci 2012; 6:304-314 10.4056/sigs.2625821 PMC3569391 23407294

[9] Lagier JC Armougom F Mishra AK Nguyen TT Raoult D Fournier PE Non-contiguous-finished genome sequence and description of *Alistipes timonensis* sp. nov. Stand Genomic Sci 2012; 6:315-324 23408657 10.4056/sigs.2685971 PMC3558960

[10] Mishra AK Lagier JC Robert C Raoult D Fournier PE Non-contiguous-finished genome sequence and description of *Clostridium senegalenses* sp. nov. Stand Genomic Sci 2012; 6:386-395 23408737 10.4056/sigs.2766062 PMC3558962

[11] Mishra AK Lagier JC Robert C Raoult D Fournier PE Non-contiguous-finished genome sequence and description of *Peptinophilus timonensis* sp. nov. Stand Genomic Sci 2012; 7:1-11 10.4056/sigs.2956294 23449949 PMC3570796

[12] Mishra AK Lagier JC Rivet R Raoult D Fournier PE Non-contiguous finished genome sequence and description of *Paenibacillus senegalensis* sp. nov. Stand Genomic Sci 2012; 7:70-81 23459006 10.4056/sigs.3056450 PMC3577113

[13] Lagier JC Gimenez G Robert C Raoult D Fournier PE Non-contiguous finished genome sequence and description of *Herbaspirillum massiliense* sp. nov. Stand Genomic Sci 2012; 7:200-209 23407294 10.4056/sigs.3086474 PMC3569391

[14] Roux V El Karkouri K Lagier JC Robert C Raoult D Non-contiguous finished genome sequence and description of *Kurthia massiliensis* sp. nov. Stand Genomic Sci 2012; 7:221-232 10.4056/sigs.3206554 23407462 PMC3569394

[15] Kokcha S Ramasamy D Lagier JC Robert C Raoult D Fournier PE Non-contiguous finished genome sequence and description of *Brevibacterium senegalense* sp. nov. Stand Genomic Sci 2012; 7:233-245 10.4056/sigs.3256677 23408786 PMC3569389

[16] Ramasamy D Kokcha S Lagier JC N’Guyen TT Raoult D Fournier PE Non-contiguous finished genome sequence and description of *Aeromicrobium massilense* sp. nov. Stand Genomic Sci 2012; 7:246-257 10.4056/sigs.3306717 23408786 PMC3569389

[17] Lagier JC Ramasamy D Rivet R Raoult D Fournier PE Non-contiguous finished genome sequence and description of *Cellulomonas massiliensis* sp. nov. Stand Genomic Sci 2012; 7:258-270 10.4056/sigs.3316719 23408774 PMC3569388

[18] Lagier JC El Karkouri K Rivet R Couderc C Raoult D Fournier PE Non-contiguous finished genome sequence and description of *Senegalemassilia anaerobia* sp. nov. Stand Genomic Sci 2013; 7:343-356 10.4056/sigs.3246665 24019984 PMC3764928

[19] Mishra AK Hugon P Lagier JC Nguyen TT Robert C Couderc C Raoult D Fournier PE Non-contiguous finished genome sequence and description of *Peptoniphilus obesi* sp. nov. Stand Genomic Sci 2013; 7:357-369 10.4056/sigs.32766871 24019985 PMC3764929

[20] Mishra AK Lagier JC Nguyen TT Raoult D Fournier PE Non-contiguous finished genome sequence and description of *Peptoniphilus senegalensis* sp. nov. Stand Genomic Sci 2013; 7:370-381 10.4056/sigs.3366764 24019986 PMC3764932

[21] Lagier JC El Karkouri K Mishra AK Robert C Raoult D Fournier PE Non-contiguous finished genome sequence and description of *Enterobacter massiliensis* sp. nov. Stand Genomic Sci 2013; 7:399-412 10.4056/sigs.3396830 24019988 PMC3764934

[22] Hugon P Ramasamy D Lagier JC Rivet R Couderc C Raoult D Fournier PE Non-contiguous finished genome sequence and description of *Alistipes obesi* sp. nov. Stand Genomic Sci 2013; 7:427-439 10.4056/sigs.3336746 24019990 PMC3764931

[23] Mishra AK Hugon P Robert C Couderc C Raoult D Fournier PE Non-contiguous finished genome sequence and description of *Peptoniphilus grossensis* sp. nov. Stand Genomic Sci 2012; 7:320-330 23408485 10.4056/sigs.3076460 PMC3569384

[24] Ezaki T Kawamura Y Li N Li ZY Zhao L Shu S Proposal of the genera Anaerococcus gen. nov., Peptoniphilus gen. nov. and Gallicola gen. nov. for members of the genus Peptostreptococcus. Int J Syst Evol Microbiol 2001; 51:1521-1528 11491354 10.1099/00207713-51-4-1521

[25] Foubert EL Douglas HC Studies on the Anaerobic Micrococci: I. Taxonomic Considerations. J Bacteriol 1948; 56:25-34 10.1128/JB.56.1.25-34.1948 18863623

[26] Murdoch DA Collins MD Willems A Hardie JM Young KA Magee JT Description of three new species of the genus Peptostreptococcus from human clinical specimens: Peptostreptococcus harei sp. nov., Peptostreptococcus ivorii sp. nov., and Peptostreptococcus octavius sp. nov. Int J Syst Bacteriol 1997; 47:781-787 10.1099/00207713-47-3-781

[27] Woese CR Kandler O Wheelis ML Towards a natural system of organisms: proposal for the domains Archae, Bacteria, and Eukarya. Proc Natl Acad Sci USA 1990; 87:4576-4579 10.1073/pnas.87.12.4576 2112744 PMC54159

[28] Gibbons NE Murray RGE Proposals Concerning the Higher Taxa of Bacteria. Int J Syst Bacteriol 1978; 28:1-6 10.1099/00207713-28-1-1

[29] Garrity GM, Holt JG. The Road Map to the Manual. In: Garrity GM, Boone DR, Castenholz RW (eds), Bergey's Manual of Systematic Bacteriology, Second Edition, Volume 1, Springer, New York, 2001, p. 119-169.

[30] Murray RGE. The Higher Taxa, or, a Place for Everything...? In: Holt JG (ed), Bergey's Manual of Systematic Bacteriology, First Edition, Volume 1, The Williams and Wilkins Co., Baltimore, 1984, p. 31-34.

[31] List of new names and new combinations previously effectively, but not validly, published. List no. 132. Int J Syst Evol Microbiol 2010; 60:469-472 10.1099/ijs.0.022855-0 20458120

[32] Rainey FA. Class II. *Clostridia* class nov. In: De Vos P, Garrity G, Jones D, Krieg NR, Ludwig W, Rainey FA, Schleifer KH, Whitman WB (eds), Bergey's Manual of Systematic Bacteriology, Second Edition, Volume 3, Springer-Verlag, New York, 2009, p. 736.

[33] Skerman VBD Sneath PHA Approved list of bacterial names. Int J Syst Bact 1980; 30:225-420 10.1099/00207713-30-1-225

[34] Prevot AR. Dictionnaire des bactéries pathogens. *In*: Hauduroy P, Ehringer G, Guillot G, Magrou J, Prevot AR, Rosset, Urbain A (*eds*). Paris, Masson, 1953, p.1-692.

[35] Ludwig W, Schleifer KH, Whitman WB. Revised road map to the phylum Firmicutes *In*: Bergey's Manual of Systematic Bacteriology, 2nd ed., vol. 3 (The Firmicutes) (P. De Vos, G. Garrity, D. Jones, N.R. Krieg, W. Ludwig, F.A. Rainey, K.-H. Schleifer, and W.B. Whitman, eds.), Springer-Verlag, New York. (2009) pp. 1-13

[36] Ezaki T Kawamura Y Li N Li ZY Zhao L Shu S Proposal of the genera *Anaerococcus* gen. nov., *Peptoniphilus* gen. nov. and *Gallicola* gen. nov. for members of the genus *Peptostreptococcus.* Int J Syst Evol Microbiol 2001; 51:1521-1528 11491354 10.1099/00207713-51-4-1521

[37] Ashburner M Ball CA Blake JA Botstein D Butler H Cherry JM Davis AP Dolinski K Dwight SS Eppig JT Gene ontology: tool for the unification of biology. The Gene Ontology Consortium. Nat Genet 2000; 25:25-29 10.1038/75556 10802651 PMC3037419

[38] Schloss PD Handelsman J Status of the microbial census. Microbiol Mol Biol Rev 2004; 68:686-691 10.1128/MMBR.68.4.686-691.2004 15590780 PMC539005

[39] Tamura K Dudley J Nei M Kumar S MEGA4: Molecular Evolutionary Genetics Analysis (MEGA) software version 4.0. Mol Biol Evol 2007; 24:1596-1599 10.1093/molbev/msm092 17488738

[40] Murdoch DA Gram-positive anaerobic cocci. Clin Microbiol Rev 1998; 11:81-120 9457430 10.1128/cmr.11.1.81 PMC121377

[41] Seng P Drancourt M Gouriet F La Scola B Fournier PE Rolain JM Raoult D Ongoing revolution in bacteriology: routine identification of bacteria by matrix-assisted laser desorption ionization time-of-flight mass spectrometry. Clin Infect Dis 2009; 49:543-551 10.1086/600885 19583519

[42] Gao S Sung WK Nagarajan N Opera: reconstructing optimal genomic scaffolds with high-throughput paired-end sequences. J Comput Biol 2011; 18:1681-1691 10.1089/cmb.2011.0170 21929371 PMC3216105

[43] Boetzer M Pirovano W Toward almost closed genomes with GapFiller. Genome Biol 2012; 13:R56 10.1186/gb-2012-13-6-r56 22731987 PMC3446322

[44] Lagesen K Hallin P Rodland EA Staerfeldt HH Rognes T Ussery DW RNAmmer: consistent and rapid annotation of ribosomal RNA genes. Nucleic Acids Res 2007; 35:3100-3108 10.1093/nar/gkm160 17452365 PMC1888812

[45] Laslett D Canback B ARAGORN, a program to detect tRNA genes and tmRNA genes in nucleotide sequences. Nucleic Acids Res 2004; 32:11-16 10.1093/nar/gkh152 14704338 PMC373265

[46] Griffiths-Jones S Bateman A Marshall M Khanna A Eddy SR Rfam: an RNA family database. Nucleic Acids Res 2003; 31:439-441 10.1093/nar/gkg006 12520045 PMC165453

[47] Punta M Coggill PC Eberhardt RY Mistry J Tate J Boursnell C Pang N Forslund K Ceric G Clements J The Pfam protein families database. Nucleic Acids Res 2012; 40:D290-D301 10.1093/nar/gkr1065 22127870 PMC3245129

[48] Hyatt D Chen GL Locascio PF Land ML Larimer FW Hauser LJ Prodigal: prokaryotic gene recognition and translation initiation site identification. BMC Bioinformatics 2010; 11:119 10.1186/1471-2105-11-119 20211023 PMC2848648

[49] Camacho C Coulouris G Avagyan V Ma N Papadopoulos J Bealer K Madden TL BLAST+: architecture and applications. BMC Bioinformatics 2009; 10:421 10.1186/1471-2105-10-421 20003500 PMC2803857

[50] Benson DA Karsch-Mizrachi I Clark K Lipman DJ Ostell J Sayers EW Gen Bank. Nucleic Acids Res 2012; 40:D48-D53 10.1093/nar/gkr1202 22144687 PMC3245039

[51] Tatusov RL Galperin MY Natale DA Koonin EV The COG database: a tool for genomoe-scale analysis of protein functions and evolution. Nucleic Acids Res 2000; 28:33-36 10.1093/nar/28.1.33 10592175 PMC102395

[52] Tatusov RL Koonin EV Lipman DJ A genomic perspective on protein families. Science 1997; 278:631-637 10.1126/science.278.5338.631 9381173

